# Measurement of femoral posterior condylar offset and posterior tibial slope in normal knees based on 3D reconstruction

**DOI:** 10.1186/s12891-021-04367-6

**Published:** 2021-05-27

**Authors:** Liangxiao Bao, Shengwei Rong, Zhanjun Shi, Jian Wang, Yang Zhang

**Affiliations:** Division of orthopaedic surgery, Department of orthopaedics, Nanfang Hospital, Southern Medical University, 1838 Guangzhou Avenue, Guangzhou, 510515 Guangdong China

**Keywords:** Femoral condylar offset, Posterior tibial slope, Total knee arthroplasty, Three dimensional reconstruction

## Abstract

**Background:**

Femoral posterior condylar offset (PCO) and posterior tibial slope (PTS) are important for postoperative range of motion after total knee arthroplasty (TKA). However, normative data of PCO and PTS and the correlation between them among healthy populations remain to be elucidated. The purpose of this study was to determine PCO and PTS in normal knees, and to identify the correlation between them.

**Methods:**

Eighty healthy volunteers were recruited. CT scans were performed followed by three-dimensional reconstruction. PCO and PTS were measured and analyzed, as well as the correlation between them.

**Results:**

PTS averaged 6.78° and 6.11°, on the medial and lateral side respectively (*P* = 0.002). Medial PCO was greater than lateral (29.2 vs. 23.8 mm, *P* <  0.001). Both medial and lateral PCO of male were larger than female. On the contrary, male medial PTS was smaller than female, while there was no significant difference of lateral PTS between genders. There was an inverse correlation between medial PCO and PTS, but not lateral.

**Conclusions:**

Significant differences exhibited between medial and lateral compartments, genders, and among individuals. An inverse correlation exists between PCO and PTS in the medial compartment. These results improve our understanding of the morphology and biomechanics of normal knees, and subsequently for optimising prosthetic design and surgical techniques.

## Background

Total knee arthroplasty (TKA) is an effective procedure for advanced disorders of the knee joint, such as osteoarthritis [1]. It is important to reconstruct anatomical alignment of the operative limb during TKA procedures, to obtain maximum range of motion (ROM) [2]. Femoral posterior condylar offset (PCO) and sagittal posterior tibial slope (PTS) are two of the most important variables during TKA procedures, utilized to determine intra-operative osteotomy.

PCO is defined as the maximum thickness of posterior condyle projecting to the tangent of the posterior cortex of femoral shaft. PTS is the postero-caudal inclination of the tibial plateau in the sagittal plane. PCO and PTS may affect ROM during flexion in different ways [3–6]. A 3-mm decrease of PCO may reduce knee flexion by 10 degrees [6], and there is an increase of 2.6 degrees of flexion with each degree of PTS [5].

In order to avoid impingement between the posterior border of the tibial plateau and femur, PCO should be restored to avoid overresecting the posterior condyle during TKA [3, 4]. An appropriate PTS provides sufficient space during knee flexion to prevent the knee joint becoming too tight [5].

There have been investigations evaluating the importance of restoring PCO and PTS after TKA [7–13]. PCO and PTS counterbalance their respective effects on ROM and dynamic stability [6]. Thus the understanding of normative data of PCO and PTS is of critical importance. However, most of the publishded studies had focused on measuring PCO and PTS in knees with osteoarthritis. Currently there are few studies exploring PCO and PTS in knees without pathologic changes [8].

In our previous study, we have identified different PTS based on different referential axes [2]. As recently reported, PCO is not restored using standard instrumentation of different manufacturers, imparing pain and functional improvements after TKA [14]. Thus the current study further focused on the variability of PCO between medial and lateral condyles, and the correlation between PCO and PTS, based on multi-slice CT scans and 3D reconstructions of knee joints of healthy volunteers. These results will be benificial to TKA prosthesis design and selection, preoperative planning, and computer-assisted surgeries [15].

## Methods

After approved by ethical committee (NFEC-2013-177) and written informed consents, healthy volunteers were recruited in this study. All the participants declared that they did not fit any of the following exclusion criteria: knee pain, deformity, abnormal movement, claudication, rheumatic fever, rickets, rheumatoid arthritis, osteoarthritis, fracture or previous surgeries. The sample size was calculated before the study, which revealed that a minimum of 61 cases was needed to establish 90% power.

CT scans and 3D reconstruction were conducted as described previously [2]. Briefly, 64-slice multi slice spiral CT scans were performed for all participants, from the femoral head to the heel of both lower limbs. Reconstructions were then performed using Mimics 10.01 software (MATERIALISE, Belgium).

**Fig. 1:**
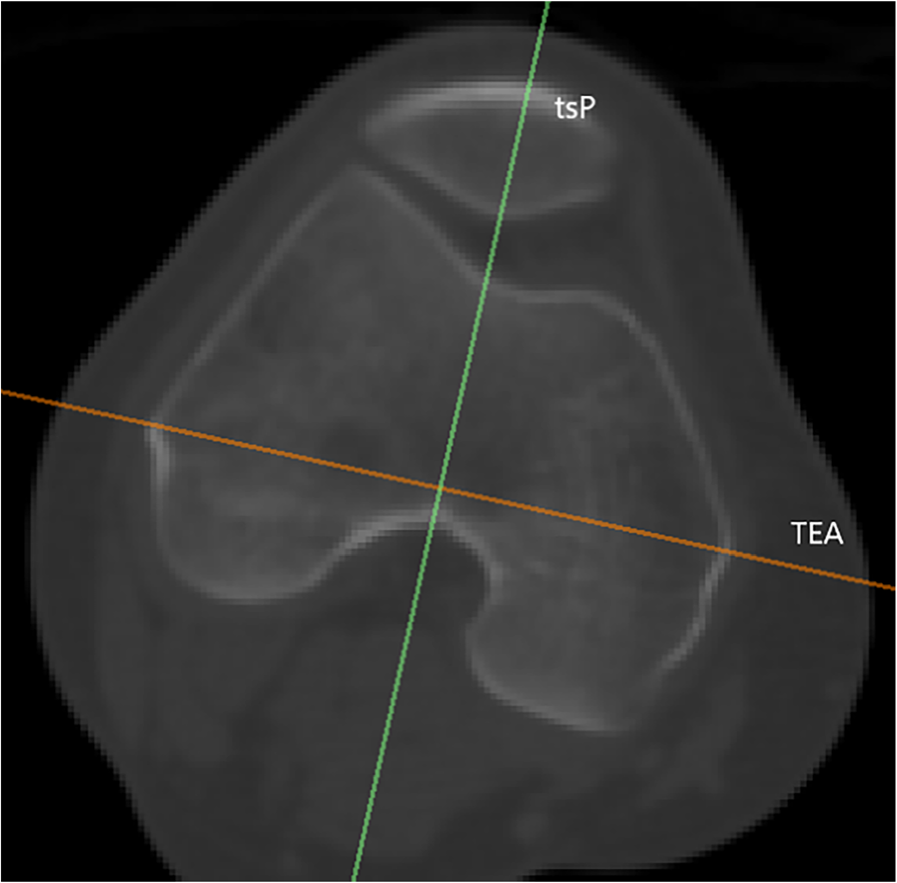
The anatomical transepicondylar axis (TEA) was defined as the straight line connecting the most prominent aspects of the medial and lateral epicondyle (orange line). The sagittal plane perpendicular to the TEA was defined as the true-sagittal plane (tsP) (green line)

**Fig. 2:**
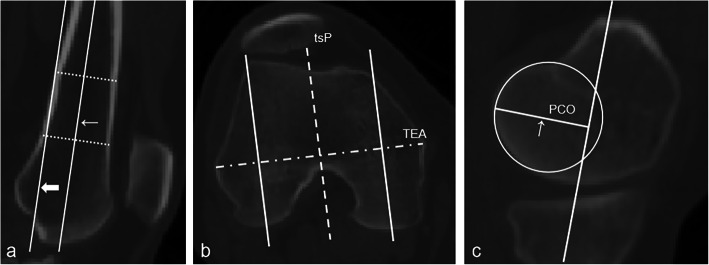
Measurement of posterior condylar offset (PCO). **a** translation of the sagittal longitudinal axis (fine arrow) to femoral posterior cortex (thick arrow). **b** translation of tsP (dotted line) alone TEA (dash-dot line). **c** PCO measurement using the largest circle fitting the peripheral border of the posterior condyle

PCO measurement was performed according to a published protocol [8]. First, transepicondylar axis (TEA) was defined as the line through the most prominent center of the femoral epicondyles in the axial plane. The true-sagittal plane (tsP) was defined as the sagittal plane perpendicular to TEA (Fig. 1) [16]. Two points located 5 cm apart, at the middle of the distal diaphyseal shaft were identified. The sagittal longitudinal axis of the femur was defined by the above two points [8], which was posteriorly shifted to tangent to posterior femoral cortex (Fig. 2a).

Then along TEA, the tsP was moved laterally to the middle of the lateral condyle, and medially to the middle of the medial condyle, on which scan PCO measurements were performed (Fig. 2b). The largest circle fitting the peripheral border of the posterior condyle was determined. PCO was then determined by the vertical distance between the translated femoral axis and the foregoing circle (Fig. 2c).

**Fig. 3:**
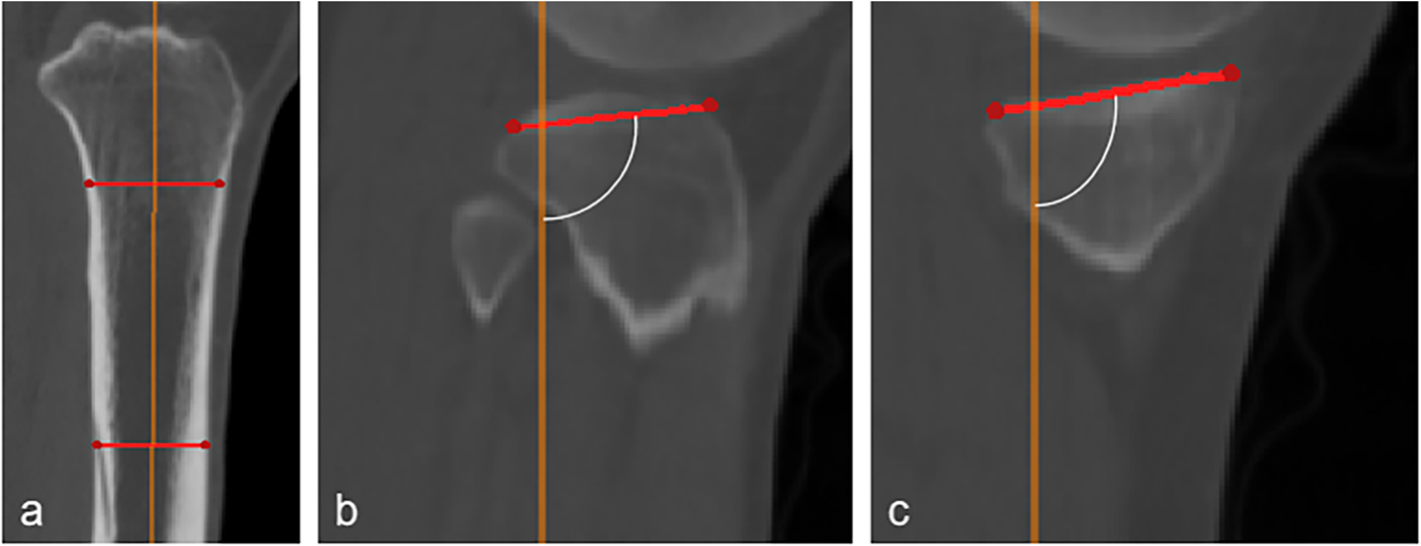
Measurement of sagittal posterior tibial slope (PTS). **a** confirmation of proximal tibial long axis (orange line) in tsP. **b** and **c** measurement of PTS, at medial and lateral side respectively

As previously described [2], for PTS measurement, the sagittal axis and the tangential line of tibial plateau were used [17] (Fig. 3). Briefly, the tangential line of tibial plateau was defined as the line passing through the center and both the anterior and posterior edge. The sagittal axis was defined as the straight line connecting midpoints of outer cortical diameter at 5 and 15 cm distal to the knee joint. Then the PTS was determined by the angle between the two lines.

Forty cases were randomly selected, in which measurement was repeated twice by two authors in 1 month. The intraclass correlation coefficient (ICC) was applied to assess the reliability: 0.00 to 0.20, poor; 0.21 to 0.40, fair; 0.41 to 0.60, moderate; 0.61to 0.80, substantial; and 0.81 to 1.00, perfect.

For statistical analysis, the data were tested for normality. Data in accordance with normal distribution were compared with paired (left and right) or unpaired (male and female) *t*-tests, otherwise non-parametric tests were applied. Pearson correlations were used to determine the relations between PCO and PTS. *P*-values< 0.05 were considered to be significant. SPSS 20.0 (IBM, USA) was used for the statistical analysis.

## Results

A total of eighty healthy volunteers (40 males and 40 females) were recruited in this study, with an average age of 31.4 (20–45) years, average height of 167.3 (151–185) cm, and average weight of 60.0 (40–80) kg.

**Fig. 4:**
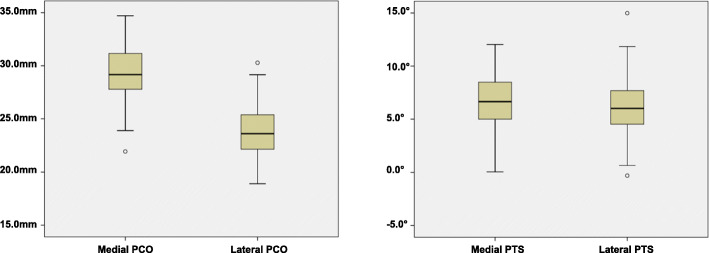
The distribution of PCO (left panel) and PTS (right panel) showing significant differences between medial and lateral compartment

There were satisfactory measurements reliability with an ICC of 0.82 for medial PCO, 0.79 for lateral PCO, 0.93 for medial PTS and 0.80 for lateral PTS. Both PCO and PTS showed a normal distribution in the medial and lateral compartments. Medial PCO and PTS were greater than lateral ones regardless of genders. The mean PCO was 29.2 mm on the medial, and 23.8 mm on the lateral side (*P* <  0.001) (Fig. 4). The mean PTS was 6.78° and 6.11° on the medial and lateral side respectively (*P* = 0.002) (Fig. 4).

There was no significant difference between left and right knee within the same individual (Table 1). Both medial and lateral PCO of male were larger than female (*P* <  0.001) (Table. 2). On the contrary, male medial PTS was smaller than female (*P* = 0.016), while there was no significant difference of lateral PTS between genders (Table 2).

**Table 1:** Measurement of PCO and PTS in normal knees

	Mean	SD	Range	95%CI	*P*-value
Medial PCO (mm)					
Left	29.4	2.49	23.9–34.7	28.8–29.9	0.390
Right	29.0	2.34	21.9–33.4	28.5–29.6	
Lateral PCO (mm)					
Left	23.5	2.12	19.8–30.3	23.0–24.0	0.074
Right	24.1	2.24	18.9–29.1	23.6–24.6	
Medial PTS (°)					
Left	6.92	2.27	2.4–12.04	6.44–7.44	0.256
Right	6.64	2.48	0.05–11.5	6.06–7.20	
Lateral PTS (°)					
Left	6.33	2.57	−0.30-14.99	5.77–6.91	0.140
Right	5.90	2.53	0.65–11.84	5.32–6.49

**Table 2:** Gender difference of PCO and PTS

	Mean	SD	Range	95%CI	*P*-value
Medial PCO (mm)					
Male	30.13	2.30	24.27–34.71	29.60–30.65	< 0.001
Female	28.27	2.15	21.94–33.03	27.81–28.74	
Lateral PCO (mm)					
Male	24.54	2.21	19.75–30.28	24.09–24.99	< 0.001
Female	23.08	1.93	18.90–26.16	22.69–23.48	
Medial PTS (°)					
Male	6.30	2.43	0.05–12.04	5.75–6.90	0.016
Female	7.22	2.24	2.77–11.50	6.74–7.75	
Lateral PTS (°)					
Male	5.92	2.38	0.65–11.84	5.37–6.51	0.375
Female	6.29	2.70	−0.30-14.99	5.66–6.90	

**Fig. 5:**
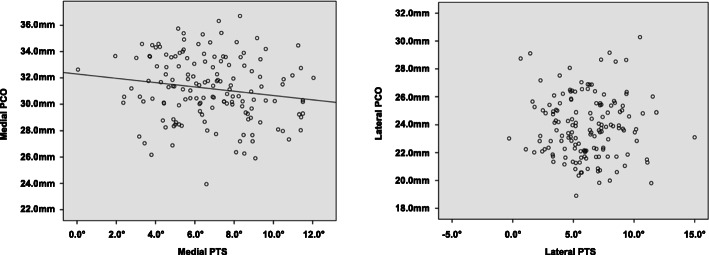
The linear correlation analysis between PCO and PTS in the medial (left panel) and lateral compartment (right panel)

In the medial compartment, an inverse correlation was detected between PCO and PTS (*r* = − 0.315; *P* = 0.026) (Fig. 5). However, no significant correlation was detected in the lateral compartment.

## Discussion

Conservation of the PCO and PTS has been regarded as a major factor to optimize ROM [6]. It was reported that decrease of PCO by 1 mm might result in a ROM reduction of 3.3–6.2° [5]. Correspondingly, each degree reduction in PTS might decrease ROM by 1.0–2.6° [4–6, 18]. Thus it is critical to understand the “normal” PCO and PTS.

PCO and PTS had been measured on conventional plain X-rays, which provides an acceptable level of accuracy under limited conditions [2]. As its imprecision can be impaired by magnification and incorrect positioning [19]. Thus, CT has been chosen as a more accurate method for measurement [20]. In our study, PCO and PTS were measured based on CT scans and 3D reconstruction, presenting differences between the medial and lateral sides which was neglected on plain radiographs [20, 21]. Further, the application of 3D reconstruction made it possible to rotate without restraint and to work in a straight forward way [2].

Gender difference has been suggested to be taken into consideration for designing knee prothesis [22]. Compared with female, male has significant greater PCO and smaller PTS in the medial compartment. As increased PCO or PTS are associated with increased ROM, and decreased PCO or PTS are associated with early tibiofemoral impingement [5], it is therefore reasonable to take this gender difference into account to obtain a maximum ROM and joint stability.

As for the relationship between medial PCO and PTS, Cinotti et al. [8] detected a significant correlation in the medial compartment but not in the lateral compartment, in magnetic resonance images. This correlation was attributed to anatomical structures: In the medial, the concave shape of the tibial plateau, the firm attachment between meniscus and tibia, and the tight medial collateral ligament, collaboratively generate a constrained articulation. While in the lateral compartment, greater laxity was exhibited due to the flatter shape of the tibial plateau, the greater mobility of the lateral meniscus and the lower tension of the lateral collateral ligament [23, 24]. However, our study demonstrated an inverse correlation between PCO and PTS in the medial compartment. Although currently we do not know the mechanism of this inverse correlation between medial PCO and PTS, the absence of meniscus during measurement, ethnic variation, and knees from healthy volunteers but not osteoarthritis patients may contribute to this finding. Consistent with our result, a previous cadaveric study [15] reported that the inverse correlation between PCO and PTS in the medial compartment, and assumed that this inverse correlation served as sagittal balance between flexion (increased PCO, increased PTS) and stability (decreased PCO, decreased PTS) [15].

PCO exhibited a variation coefficient of 8.3 and 9.2% on the medial and lateral side. And PTS showed a variation coefficient of 35.0 and 41.7% on the medial and lateral side, with the extreme values being − 0.3°and 14.99° respectively. This implies the complex anatomy features and asymmetric articular surface geometry of the knee which is necessary for physiologic motions [25, 26]. During TKA precudures, PCO should be conserved as much as possible to avoid an unbalanced knee in flexion. However, there is a trend in over-resection of the medial condyle and the under-resection of the lateral posterior condyle [14]. Thus the sagittal tibial cut during TKA should be performed parallel to the intrinsic tibial slope [6] but not perpendicular to the tibial sagittal mechanical axis [25] in patients with pronounced PCO to avoid a tight knee in flexion [15]. Further, current prosthetic design of the knee universally ignores the asymmetry articular surface, which may be the reasons for non-physiologic, paradoxical kinematics [26, 27]. Introducing asymmetry may allow advantages to control sagittal plane motion, thus the medial-pivot concept has been proved to have many advantages [28]. The medial-pivot TKA is characterized by equal radius of distal and posterior femoral condylar, and asymmetrical polyethylene insert [29]. Constraints on the medial side of the insert cause the medial compartment to act a pivot during movement in the sagittal plane. Thus, when the knee flexes, posterior translation of the femur is restricted to the unconstrained lateral compartment [29]. Clinically, according to a published meta-analysis, the pivot system presents better longevity and outcome than other systems [30].

The present study has several limitations: First, only volunteers from South China were included, whose measurements can not be widely generalized to all populations. However, these results will be useful to study ethnic variations. Second, the subjects were from young population (20–45 years old), rather than a wide range of ages. For this reason, these data were not influenced by pathologically altered bone (such as age-related degeneration) and help understanding kinematics of normal knees.

## Conclusions

This study reports normative data of PCO and PTS in the knee joint. Differences were exhibited between medial and lateral compartments, genders, and among individuals. An inverse correlation exists between PCO and PTS in the medial compartment, probably serving as sagittal balance between flexion and stability. The results of this study improve our understanding of the morphology of normal knees. This is important for better understanding the biomechanics of the knee, and subsequently for optimising prosthetic design and surgical techniques. These represent potential areas for future research.

## Data Availability

The datasets during and/or analysed during the current study are available from the corresponding author on reasonable request.

## Abbreviations

PCO: Posterior condylar offset
PTS: Posterior tibial slope
TKA: Total knee arthroplasty
ROM: Range of motion
TEA: Transepicondylar axis
tsP: True-sagittal plane
ICC: Intraclass correlation coefficient
